# Simple Changes to Emergency Department Workflow Improve Analgesia in Mechanically Ventilated Patients

**DOI:** 10.5811/westjem.2018.4.36879

**Published:** 2018-05-16

**Authors:** Derek L. Isenberg, Katrina M. Kissman, Ellie P. Salinski, Mark A. Saks, Loreen B. Evans

**Affiliations:** * Lewis Katz School of Medicine at Temple University, Department of Emergency Medicine, Philadelphia, Pennsylvania; † Crozer Chester Medical Center, Department of Emergency Medicine, Upland, Pennsylvania

## Abstract

**Introduction:**

In 2013 the Society for Critical Care Medicine (SCCM) published guidelines for the management of pain and agitation in the intensive care unit (ICU). These guidelines recommend using an analgesia-first strategy in mechanically ventilated patients as well as reducing the use of benzodiazepines. Benzodiazepines increase delirium in ICU patients thereby increasing ICU length of stay. We sought to determine whether a simple educational intervention for emergency department (ED) staff, as well as two simple changes in workflow, would improve adherence to the SCCM guidelines.

**Methods:**

This was a cohort study that took place from 2014–2016. All patients who were intubated in the ED by an emergency physician (EP) during this time were eligible for inclusion in this study. In January 2015, we began an educational campaign with the ED staff consisting of a series of presentations and online trainings. The impetus for our educational campaign was to have best practices in place for our new emergency medicine residency program starting in July 2016. We made two minor changes in our ED workflow to support this educational objective. First, fentanyl infusions were stocked in the ED. Second, we instituted a medication order set for mechanically ventilated patients. This order set nudged EPs to choose medications consistent with the SCCM guidelines. We then evaluated the use of opioids and benzodiazepines in mechanically ventilated patients from 2014 through 2016 using Fisher’s exact test. All analyses were conducted in the overall sample (n=509) as well as in subgroups after excluding patients with seizures/status epilepticus as their primary admission diagnosis (n=461).

**Results:**

In 2014 prior to the interventions, 41% of mechanically ventilated patients received an opioid, either as an intravenous (IV) push or IV infusion. In 2015 immediately after the intervention, 71% of patients received an opioid and 64% received an opioid in 2016. The use of benzodiazepine infusions decreased from 22% in 2014 to 7% in 2015 to 1% in 2016.

**Conclusion:**

A brief educational intervention along with two simple changes in ED workflow can improve compliance with the SCCM guidelines for the management of pain and agitation in mechanically ventilated patients.

## INTRODUCTION

### Background

In 2013 the Society for Critical Care Medicine (SCCM) published its Clinical Practice Guidelines for the Management of Pain, Agitation, and Delirium in Adult Patients in the Intensive Care Unit (ICU).^1^ These guidelines disseminated best practices in the care of both critically ill and mechanically ventilated patients. The SCCM Guidelines recommend that “intravenous (IV) opioids be considered as the first-line drug class of choice to treat non-neuropathic pain in critically ill patients.” For sedation, the SCCM guidelines suggest that “sedation strategies using nonbenzodiazepine sedatives may be preferred over sedation with benzodiazepines (either midazolam or lorazepam) to improve clinical outcomes in mechanically ventilated adult ICU patients. Benzodiazepines have been showed to increase ICU delirium thereby increasing ventilator days and ICU length of stay (LOS).^2^ ICU delirium is well known to increase ICU and hospital LOS as well as six-month mortality.^3^

Mechanically ventilated patients are subjected to many painful procedures such as urinary catheters, central venous access lines, and frequent blood draws. Simply having an endotracheal tube in place is painful. By treating pain first, the SCCM guidelines aim to increase patient comfort while simultaneously reducing the occurrence of delirium in the ICU. Prior studies that have looked at the emergency department (ED) treatment of post-intubation patients found suboptimal use of analgesic and anxiolytic medications. For example, Bonomo in 2007 found that 33% of mechanically ventilated patients in the ED received no anxiolytics, 53% received no analgesia, and 20% received neither analgesia nor anxiolytics.^4^ Additionally, a large study using the National Ambulatory Medical Care Survey found that less than 50% of mechanically ventilated patients received a sedative or opioid medication.^5^

In 2015 the Accreditation Council for Graduate Medical Education Residency Review Committee approved a new emergency medicine (EM) residency at our institution. In preparation for the new residency, we undertook an assessment of our current clinical practices, seeking to have best practices in place for the new residency program. Therefore, we sought to determine whether a brief educational intervention coupled with two simple changes in ED workflow would improve adherence to the SCCM guidelines. More specifically, we wanted to increase the use of opioids and decrease the use of benzodiazepines in mechanically ventilated patients in the ED.

## METHODS

### Design

This was a cohort study that took place from 2014–2016 at Crozer Chester Medical Center (CCMC), a community-based 300-bed tertiary care center, Level II trauma center, and regional burn center. CCMC has multiple graduate medical programs and initiated an EM residency program in July 2016. The CCMC ED treats approximately 53,000 patients per year with an admission rate of approximately 36%.

### Patients

All patients who were intubated in the ED by emergency physicians (EPs) between January 1, 2014, and December 31, 2016, were eligible for inclusion. We identified all intubated patients through retrospective review of our electronic medical record (EMR) (Optum ED PulseCheck®, Optum Clinical Solutions, Inc. Eden Prairie, Minnesota). Trauma patients were excluded from our study because, in our facility, these patients were intubated by anesthesia with subsequent medication management by the trauma team. Other exclusions included children less than the age of 18, intubated patients who died in the ED, patients who were intubated solely for a procedure and then extubated (such as endoscopy), or patients who were transferred out of the hospital system. We excluded the latter patients because the receiving facilities often requested a specific sedation and analgesia package for transport. Finally, we excluded any patients who were intubated by the authors of this study as they were aware of its hypothesis.

**Population Health Research Capsule**

What do we already know about this issue?

Analgesia provided to mechanically ventilated patients in the emergency department (ED) is often inadequate and does not follow published recommendations.

What was the research question?

Can simple changes in ED workflow improve the use of analgesia in mechanically ventilated patients?

What was the major finding of the study?

Simple changes to the electronic medical record and stocking fentanyl infusions in the ED increase use of analgesia in intubated patients.

How does this improve population health?

Simple workflow changes that encourage following published guidelines can change physician behavior and potentially lead to improved patient outcomes.

For all patients who met inclusion criteria, data was extracted via chart review and entered into a Microsoft Excel (Redmond, Washington) database in a blinded fashion for review and analysis. We considered the time period February 1, 2014, through December 31, 2014 our “pre-intervention” period. We used January 2015 as a “wash out” period in which ED staff and physicians were acclimated to the new analgesia-first strategy. We gathered our outcomes data from February 1, 2015, until December 31, 2016.

### Interventions

In January 2015 the authors began an educational campaign to improve sedation and analgesia practices for mechanically ventilated patients. First, to educate the EPs we gave brief presentations at two consecutive faculty meetings. We reviewed the SCCM guidelines, discussed our current sedation and analgesia practices, and made recommendations as to the appropriate medications for mechanically ventilated patients. We also sent periodic educational emails to the faculty. To educate the nursing staff, we provided a similar, brief, 20-minute educational online presentation using Brainshark© (Waltham, Massachusetts). In addition, we met with the nurses at their daily shift huddles to discuss the new initiative.

**Figure 1:**
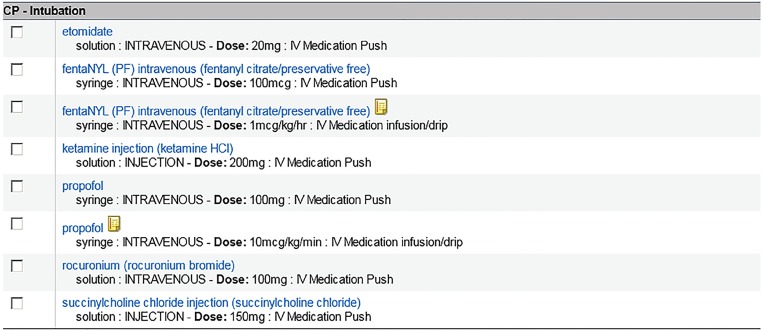
Intubation medication order set (Picis Clinical Solutions© Wakefield, Massachusetts).

To support our new initiative, we made two changes in our ED workflow. First, fentanyl infusions were stocked in the ED medication-dispensing machines. This change allowed nurses to access fentanyl infusions at the time of intubation rather than waiting on infusions to be prepared in and delivered from the central pharmacy (which had been the standard practice). Secondly, we instituted a best-practices order set for mechanically ventilated patients. As shown in Figure 1, EPs could choose from pre-populated medication choices that included fentanyl and propofol. EPs could still order benzodiazepines but had to use a search function in the EMR.

### Outcomes

Our primary outcome was the percentage of mechanically ventilated patients who received an opioid. Secondary outcomes included the percentage of mechanically ventilated patients who received any benzodiazepine and the percentage of patients who received no sedation. We also performed a subgroup analysis excluding patients with a primary diagnosis of seizure/status epilepticus as benzodiazepines may be the most appropriate medications for these patients.

### Statistical Analysis

We used descriptive statistics to characterize the use of opioids and benzodiazepines in this sample. Continuous variables were described with means, standard deviations, and ranges, and categorical variables were described with frequencies and percentages. Changes in the use of opioids and benzodiazepines in patients from 2014 vs. 2015, 2014 vs. 2016, and 2015 vs. 2016 were evaluated using Fisher’s exact test. We conducted analyses in the overall sample (n=509), as well as in a subgroup excluding patients with seizures or status epilepticus as their primary admission diagnosis (n=461). Statistical significance was taken at the 0.05 level. No adjustments were made to account for multiplicity. This study was approved by the investigational review board of CCMC.

## RESULTS

### Overall Sample

**Figure 2:**
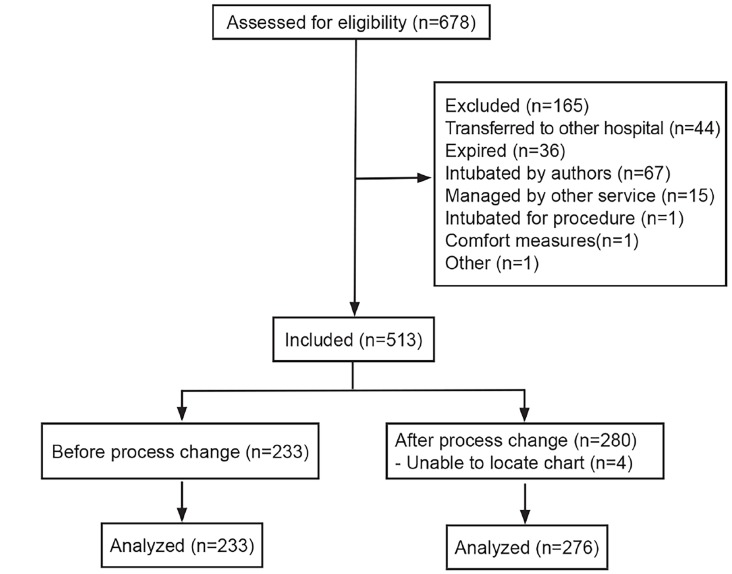
CONSORT (Consolidated Standards of Reporting Trials) flow diagram.

We included in the study 509 patients who were mechanically ventilated (Figure 2). Of the 509 total patients, we obtained data from 233 patients in 2014, 150 in 2015, and 126 in 2016. Patient demographics for the overall sample are summarized in Table 1.

The use of opioids and benzodiazepines in the overall sample from 2014–2016 is summarized in Table 2. In 2014, prior to the workflow changes, 41% of mechanically ventilated patients received an opioid, either as an intravenous push (IVP) or as an IV infusion (n=95). In 2015, immediately after the intervention, and in 2016, the later study period, 71% (n=106) and 64% (n=81) of mechanically ventilated patients received an opioid (both p<0.0001). We found significant differences in the percent of patients receiving an opioid IV infusion in 2014 vs. 2015 and 2014 vs. 2016 (both p<0.0001). Specifically, only 29% (n=67) of mechanically ventilated patients received an opioid IV infusion in 2014 compared to 61% (n=92) in 2015 and 61% (n=77) in 2016.

The use of benzodiazepine infusions significantly differed in 2014 vs. 2015 and 2014 vs. 2016 (both p<0.0001). Specifically, the use of benzodiazepine infusions was 22% (n=52) in 2014, 7% (n=10) in 2015, and 1% (n=1) in 2016. Additionally, significant differences were found in the percent of patients receiving any benzodiazepine, either as an IVP or infusion, in 2014 vs. 2015, and 2014 vs. 2016 (both p<0.0001). Sixty-two percent (n=144) of mechanically ventilated patients received a benzodiazepine in 2014, compared to 34% (n=50) in 2015 and 29% (n=37) in 2016. There were no significant differences in the percent of patients receiving propofol or no sedation/analgesia in 2014 vs. 2015 and 2014 vs. 2016.

### Subgroup Analysis

We also conducted Fisher’s exact tests in a subgroup that excluded patients with seizures or status epilepticus as their primary admission diagnosis. A total of 461 patients were used in this subgroup analysis, with 211 patients in 2014, 135 in 2015, and 115 in 2016. Similar results were seen in this subgroup of patients. Table 3 summarizes the use of opioids and benzodiazepines in this subgroup from 2014–2016. In 2014, 41% (n=87) of mechanically ventilated patients received an opioid, either as an IVP or an IV infusion, compared to 71% (n=96) in 2015, and 65% (n=75) in 2016 (both p<0.0001). Significant differences were found in the percent of patients receiving an opioid infusion in 2014 vs. 2015 and 2014 vs. 2016 (both p<0.0001). Specifically, 29% (n=61) of patients received an opioid infusion in 2014, compared to 42% (n=84) in 2015, and 63% (n=72) in 2016.

The use of benzodiazepine infusions significantly differed in 2014 vs. 2015, and in 2014 vs. 2016 (both p<0.0001). Specifically, the use of benzodiazepine infusions was 16% (n=34) in 2014, 7% (n=9) in 2015, and 1% (n=1) in 2016. Additionally, there were significant reductions in the percent of patients receiving any benzodiazepine in 2014 vs. 2015 and 2014 vs. 2016 (both p<0.0001). Sixty-seven percent (n=127) of patients received a benzodiazepine in 2014, compared to 33% (n=43) in 2015, and 26% (n=30) in 2016. No significant differences were found in the percent of patients receiving propofol or no sedation/analgesia in 2014 vs. 2015 and 2014 vs. 2016.

## DISCUSSION

Although the SCCM guidelines are largely directed toward ICU care, we believe these recommendations should be adopted for mechanically ventilated patients in the ED to provide a unified care strategy.^1^ As a result of our interventions, we were able to significantly increase the use of opioids in mechanically ventilated patients while simultaneously decreasing the use of benzodiazepines. We were able to effect this change in medication ordering while maintaining the overall percentage of patients who received analgesia and/or sedation following mechanical ventilation at 82–83%, significantly above reported rates.^4^,^5^ As follow up to this research, we are currently evaluating whether the increased use of an analgesia-first strategy in the ED reduces ventilator LOS in mechanically ventilated patients.

In the current study, we failed to observe a change in the total number of patients who did not receive analgesia or sedation following intubation over the three-year study period. We suspect this is due to a subset of patients who require no sedation or analgesia while on the ventilator. For example, the patient with a devastating intracranial hemorrhage may not require sedation or analgesia. Similarly, a patient with a depressed mental status from an opioid, benzodiazepine, or polysubstance ingestion may not require sedation or analgesia in the initial hours after initiation of mechanical ventilation.

With the advent of the EMR, clinical support tools have been embedded into the system as a way to improve resource utilization. In 2005 Samore et al. tested the use of an electronic decision aid for primary care providers to prescribe antibiotics for acute respiratory tract infections.^6^ The authors were able to reduce antibiotic prescriptions by 8.8% in the intervention group that used the decision aid compared to the control group.

Additional research involving the integration of clinical support tools in the ED EMR has focused on decreasing inappropriate imaging. Gupta et al. showed that a decision support tool for mild traumatic head injury improved compliance with published guidelines by 27%.^14^ An embedded support tool for the ordering of computed tomography (CT) pulmonary angiograms decreased ordering from 2.6% to 2.1%, while increasing the positive yield of the studies from 5.8% to 9.8%.^8^

Most recently, Heitz et al. conducted a trial of EMR-embedded clinical support tools to reduce inappropriate imaging for head trauma, cervical spine injuries, and pulmonary embolism. This study of 235,858 ED visits found that the embedded support tools reduced the ordering of brain CTs by 10%, cervical spine CTs by 6% and pulmonary embolism studies by a non-significant 2%. Interestingly, although the most-frequent users of CT decreased their use, some of the least-frequent users increased their use of CT.

Our study is one of the first to look at EMR-embedded clinical support for prescribing practices in the ED. Rather than a series of checkboxes or pop-up menus, which are typically used in EMR-embedded clinical support tools, we used a principle called “nudging” to push the emergency physician (EP) toward choosing opioids and non-benzodiazepine medications for sedation.^10^ This study supports the idea that simple changes in the EMR workflow can nudge EPs toward certain medication order choices. We hope that future research will continue to examine how redesign of ED workflow, specifically the EMR, can aid EPs in selecting the best medication choices for their patients.

## LIMITATIONS

As the data was gathered retrospectively, we have the standard limitations of a chart review. For example, if an intubation procedure was not properly recorded in the EMR, that patient would not have been included in the study for analysis. We also only evaluated a subset of mechanically ventilated patients in our ED as we did not include in our study trauma patients, transfers out of the system, or children. In addition, only medications ordered through the EMR were included. It is always possible that medications were given after a verbal order and not later recorded in the EMR.

## CONCLUSION

In summary, a brief educational intervention and two simple changes in ED workflow – stocking fentanyl infusions in the ED and redesigning the medication ordering screen – can improve compliance with the SCCM guidelines for the management of pain and sedation in mechanically ventilated patients. This study also supports the idea that the EMR can function as a clinical support tool to nudge physicians to improve medication ordering practices.

## Figures and Tables

**Table 1:** Demographics for all mechanically ventilated patients from 2014–2016 (n=509).

	2014 n=233	2015 n=150	2016 n=126
Female [n (%)]	110 (47%)	57 (38%)	63 (50%)
Age (years)			
Mean (SD)	54.1 (18.7)	55.3 (18.3)	54.5 (19.5)
Range	18–92	18–94	18–94
Reasons for intubation			
Cardiac	21	13	13
Change in mental status	19	6	5
GI bleed	7	2	3
Other	17	7	24
Overdose	37	37	15
Respiratory	82	51	34
Seizure/status epilepticus	22	15	13
Sepsis	18	11	12
Stroke	10	8	7

*SD*, standard deviation; *GI*, gastrointestinal.

**Table 2:** Use of opioids and benzodiazepines in all mechanically ventilated patients from 2014–2016 (n=509).

	2014 n=233	2015 n=150	P value	2014 n=233	2016 n=126	P value
Received opioid IVP [n (%)]	76 (33%)	62 (41%)	0.1028	76 (33%)	51 (40%)	0.1651
Received opioid IV infusion [n (%)]	67 (29%)	92 (61%)	<0.0001	67 (29%)	77 (61%)	<0.0001
Received any Opioid [n (%)]	95 (41%)	106 (71%)	<0.0001	95 (41%)	81 (64%)	<0.0001
Received benzodiazepine IVP [n (%)]	137 (59%)	48 (32%)	<0.0001	137 (59%)	37 (29%)	<0.0001
Received benzodiazepine infusion [n (%)]	52 (22%)	10 (7%)	<0.0001	52 (22%)	1 (1%)	<0.0001
Received any benzodiazepine [n (%)]	144 (62%)	50 (34%)	<0.0001	144 (62%)	37 (29%)	<0.0001
Received propofol [n (%)]	79 (34%)	48 (32%)	0.5100	79 (34%)	47 (37%)	0.7100
Received propofol only [n (%)]	10 (4%)	5 (3%)	0.6300	10 (4%)	10 (8%)	0.0200
No sedation [n (%)]	45 (19%)	23 (15%)	0.2000	45 (19%)	23 (18%)	0.7700

*IVP*, intravenous push, *IV,* intravenous.

**Table 3:** Use of opioids and benzodiazepines in non-seizure patients from 2014–2016 (n=461).

	2014 n=211	2015 n=135	P value	2014 n=211	2016 n=115	P value
Received opioid IVP [n (%)]	69 (33%)	56 (41%)	0.0873	69 (33%)	48 (42%)	0.0873
Received opioid IV infusion [n (%)]	61 (29%)	84 (42)	<0.0001	61 (29%)	72 (63%)	<0.0001
Received any opioid [n (%)]	87 (41%)	96 (71%)	<0.0001	87 (41%)	75 (65%)	<0.0001
Received benzodiazepine IVP [n (%)]	120 (57%)	41 (30%)	<0.0001	120 (57%)	30 (26%)	<0.0001
Received benzodiazepine infusion [n (%)]	34 (16%)	9 (7%)	<0.0001	34 (16%)	1 (1%)	<0.0001
Received any benzodiazepine [n (%)]	127 (60%)	43 (33%)	<0.0001	127 (60%)	30 (26%)	<0.0001
Received propofol [n (%)]	68 (32%)	36 (27%)	0.2400	68 (32%)	31 (36%)	0.3600
Received propofol only [n (%)]	10 (5%)	4 (3%)	0.2800	10 (5%)	9 (8%)	0.1400
No sedation [n (%)]	42 (20%)	23(17%)	0.3800	42 (20%)	23 (20%)	0.1000

*IVP*, intravenous push, *IV,* intravenous.
